# Effects of Raga music and Chinese five-element on milk production, antioxidant, neuroendocrine, immune, and welfare indicators in dairy cows

**DOI:** 10.3389/fvets.2025.1623026

**Published:** 2025-06-30

**Authors:** Zongjing Cao, Huiqiu Zhao, Zhihua Feng, Bowen Yang, Zhijia Li, Xiuguo Ma, Sukun Gu, Ning Ma

**Affiliations:** ^1^College of Animal Science and Technology, Hebei Agricultural University, Baoding, China; ^2^Hebei Provincial Animal Husbandry General Station, Shijiazhuang, China; ^3^College of Veterinary Medicine, Hebei Agricultural University, Baoding, China

**Keywords:** dairy cows, music, milk production, antioxidant, neuroendocrine, welfare

## Abstract

In recent years, increasing attention has been paid to the effects of music on animal productivity. However, research specifically examining music’s impact on dairy cows remains limited, with existing studies reporting inconsistent findings. This study investigated the effects of Raga music and Chinese Five-Element music on production performance, stress response, neuroendocrine function, immune system, and welfare indicators in lactating dairy cows. Sixty healthy lactating Holstein cows with similar parity were randomly divided into three treatment groups, with 20 replicates in each group. The experiment involved three feeding environments (no music/classical music/traditional Chinese five-element music) and lasted for 60 days. Results showed that compared with the control group, dairy cows receiving music therapy exhibited a significant increase in average daily milk yield (*p* < 0.001). Both music intervention treatments had lower feed-to-milk ratios than the control group, with the Raga music treatment demonstrating the lowest ratio (*p* > 0.05). Dairy cows in the music intervention groups displayed significant improvements in serum biochemical parameters, characterized by decreased levels of alanine aminotransferase (ALT) and triglycerides (TG) (*p* < 0.05), and increased concentrations of glucose (GLU) and serum albumin (ALB) (*p* < 0.05). In antioxidant indices, enhanced glutathione peroxidase (GSH-PX) activity (*p* < 0.001) and reduced malondialdehyde (MDA) levels (*p* < 0.001) were observed. Notably, music intervention significantly increased serum concentrations of immunoglobulins G (IgG), M (IgM), and A (IgA) (*p* < 0.05), suggesting its potential role in enhancing immune function in dairy cows. Dairy cows exposed to Raga music showed significant neuroendocrine and behavioral changes, with decreased levels of glutamate (Glu) and cortisol (COR) (*p* < 0.05), and increased concentrations of growth hormone (GH), 5-hydroxytryptamine (5-HT), β-endorphin (β-EP), substance P (SP), and γ-aminobutyric acid (GABA) (*p* < 0.05). Behaviorally, cows in the experimental groups exhibited reduced physical activity (*p* < 0.05) but increased rumination and lying time (*p* > 0.05), indicating improved relaxation and metabolic efficiency. In conclusion, the comprehensive assessment demonstrates that music intervention significantly enhances milk production, antioxidant capacity, immune function, and overall welfare in dairy cows. Among the interventions, Raga music exhibits the most pronounced effects, particularly in achieving a lower feed-to-milk ratio.

## 1 Introduction

Music, as an auditory stimulus, has demonstrated the capacity to elicit physiological and biochemical responses through the propagation of sound waves (1). The potential of music in treating secondary diseases has garnered significant attention, particularly for conditions like Alzheimer’s and acute diseases pain (2). Studies have shown combining music and oxytocin massage can significantly boost lactation and reduce maternal anxiety (3). Moreover, the rhythmic qualities of music have the potential to impact physiological rhythms and psychological equilibrium, thereby fostering overall well-being (4). Uplifting music tends to evoke positive emotion (5), and music therapy has been substantiated as an effective intervention for alleviating anxiety in postpartum women (6).

Providing music can enhance the welfare of laboratory animals by contributing to environmental enrichment, alleviating stress, and promoting behavioral modification (7). Studies have shown that exposure to music reduces stress-related behaviors (such as vocalizations) in animals, suggesting that music has a regulatory effect on the emotional states of animals (8). In animal studies, music has been found to enhance synaptic plasticity and perceptual behaviors in chicks (9). Additionally, it has been shown that slower musical compositions extend the resting duration of pigs, whereas fast, loud music can provoke aggression and diminish immune (10, 11). Music intervention can improve egg production and hatchability in quails (12). Furthermore, it has been found to reduce stress levels in broilers raised in high-density conditions (13).

In current research on dairy cows, music intervention can increase milk production while shortening milking time (14), and elevate hormone levels related to milk production in cows (15). In fully automated milking systems, the application of music can effectively reduce cow stress and shorten milking time (16).

However, while the benefits of music on animal welfare and productivity are increasingly recognized, these benefits are often species-specific and dependent on the type of music played (1). The use of Raga and Chinese five-element music in dairy cow production has not been studied. In Indian classical music, the melodic system known as Raga typically begins at 50–60 beats per minute (BPM) and gradually accelerates to 100–130 BPM in its main section, employing complex rhythmic cycles. Performance durations range from as short as 5 min to as long as 2 h, representing the traditional soul and essence of this art form. Academic studies have demonstrated that Raga can elicit positive emotional responses in listeners (17). Playing Raga music can reduce milking time and increase speed while lowering cortisol levels in the serum of dairy cows (18). Chinese five-element music, rooted in the theories of yin-yang and the five musical scales (jué, zhèng, dìng, shāng, yīn), has traditionally been employed for treating various diseases. It typically features a tempo of 60–110 (BPM) and a single-track duration of 3–5 min. Recent studies have demonstrated that test subjects receiving this traditional musical therapy exhibited lower cortisol levels and alleviated depressive symptoms, suggesting its potential efficacy as a therapeutic intervention (19).

There have been no reported studies on the effects of playing these two types of music on lactating cows. Therefore, the present study aimed to evaluate the impacts of two distinct music genres, raga music and Chinese five-element music, on the production performance, serum biochemical indices, anti-stress indicators, neuroendocrine parameters, immune function, and welfare metrics of dairy cows over an extended period. Through a controlled trial, this study systematically compared these effects to screen for the music type more suitable for dairy cows.

## 2 Materials and methods

### 2.1 Source of animals and experimental design

The experiment was carried out on dairy cows at the Xingtai Branch Cattle Farm of Beijing Shounong Animal Husbandry. A total of 60 healthy lactating Holstein cows with 3–4 parities and similar lactation days (150–180 days) were selected and divided into 3 groups (20 cows per group), with each cow serving as an independent experimental unit. Feeding staff, sample collection and experiment personnel, and data analysis personnel were all unaware of the groups to which the treated cows belonged, in order to avoid bias. Cows in the control group were not exposed to music, whereas those in the experimental groups were exposed to two types of pure-tone music: ‘Raga’ and ‘Chinese five-element’ music (see Supplementary material for details). The trial lasted for 67 days, including a 7-day pre-test period and a 60-day experimental period. After feeding at 05:00, 13:00, and 21:00 daily, music was played for 0.5 h per session, similarly escalating to 1.5 h over time. During the formal experimental period, music was ensured to be played for 9 h daily. Prior to the experiment, ambient noise levels in all testing environments were measured to standardize acoustic conditions across groups. Music volume was maintained at 65–75 dB without external noise interference. Three barns located more than 200 meters apart were utilized to prevent sound interference between groups, with music players spaced at five-meter intervals for even distribution.

### 2.2 Experimental diets and feeding management

The basal diet was formulated in accordance with NRC (2001) guidelines (the main components of the basic diet are available in Supplementary Table 2). Diets formulated to meet nutritional requirements were provided ad libitum to all groups. Cows were fed three times daily (05:00, 13:00, and 21:00), and four cleaned drinking troughs per barn ensured access to fresh water.

### 2.3 Milk production, feed intake, and milk quality

Throughout the trial, daily milk yield was automatically recorded via the milking system. Milking times were set at 04:00, 12:00, and 20:00 daily. Total feed intake and leftovers were measured daily to calculate average feed consumption. On days 0, 30, and 60, 100 mL milk samples were collected in the morning, midday, and evening and placed into three test tubes. Samples collected three times daily were mixed at a 40:30:30 ratio, preserved with potassium dichromate, and stored at −20°C. Analyzed indicators included fat, protein, lactose, somatic cell count (SCC), total solids, and solids-not-fat. Detection was performed using a CombiScope FTIR 600HP in accordance with the standard (GB19301-2010).

### 2.4 Serum biochemical indices

Blood samples (10 mL) were collected from the caudal vein of all experimental cows on day 30 and day 60. Samples were allowed to clot at room temperature, refrigerated at 4°C for 30 min, and then centrifuged at 3,500 × g for 15 min. The supernatant was transferred to 1.5 mL microcentrifuge tubes and stored at −20°C until analysis. Serum biochemical indices, including albumin (ALB, NO. A028-1-1), glucose (GLU, NO. A154-1-1), triglyceride (TG, NO. A110-1-1), alanine aminotransferase (ALT, NO. A042-1-1), and non-esterified fatty acids (NEFA, NO. A042-1-1), were measured using commercial kits according to the manufacturer’s protocols. All kits were provided by Nanjing Jiancheng Bioengineering Institute (Nanjing, China).

### 2.5 Antioxidant index

The levels of total antioxidant capacity (T-AOC, NO. BC1170), glutathione peroxidase (GSH-PX, NO. BC0175 and BC0200), and malondialdehyde (MDA, NO. BC0025) were determined using kits from Beijing Solarbio Science & Technology Co., Ltd.

### 2.6 Immunologic function

The detection kits for immunoglobulin A (IgA, NO. A088-2-1), immunoglobulin G (IgG, NO. A089-2-1), and immunoglobulin M (IgM, NO. A090-2-1) were used to quantify the concentrations according to the manufacturer’s protocols. The kits were obtained from a commercial supplier in Nanjing, Jiangsu, China.

### 2.7 Neuroendocrine index

Serum neuroendocrine markers, including 5-hydroxytryptamine (5-HT, NO. A085-2-1), γ-aminobutyric acid (GABA, NO. MR6120), cortisol (COR, NO. MR6080), growth hormone (GH, NO. MR6095), β-endorphin (β-EP, NO. HMY007-96 T), and substance P (SP, NO. DY314), were analyzed using an r-911 radioimmunoassay analyzer in combination with a Mindray BS2800M biochemical analyzer.

### 2.8 Welfare index

Animal welfare parameters (lying time, rumination duration, activity levels) were continuously monitored for 24 h using Allflex accelerometer collars, with data recorded in minute intervals.

### 2.9 Statistical analysis

All statistical analyses were performed using IBM SPSS Statistics Version 26.0 (IBM Corp.). Data were first assessed for normality via the Kolmogorov–Smirnov (K-S) test, followed by one-way analysis of variance (one-way ANOVA) to examine intergroup differences. For significant ANOVA results (*p* < 0.05), *post hoc* comparisons were conducted using Duncan’s multiple range test (exploratory analyses) and the Benjamini-Hochberg (FDR) correction (hypothesis-driven analyses), with results presented as mean ± standard deviation. Primary outcomes were defined as statistically significant at *p* < 0.05 (FDR-corrected *q* < 0.10).

## 3 Results

### 3.1 Milk production and feed intake and feed to milk ratio

As shown in Table 1, cows exposed to either Raga or Chinese five-element music produced significantly more milk (*p* < 0.05) than control cows. However, both music intervention treatment demonstrated improved feed efficiency, as indicated by a lower feed-to-milk ratio (*p* < 0.05), compared with the control group.

**Table 1:** Effect of Raga and Chinese five-element music on milk production and feed intake (kg) and feed to milk ratio (%).

Items	Time/d	Control	Raga	Chinese five-element	*P*
Milk production	0–30	36.45 ± 1.59^b^	37.13 ± 2.65^a^	36.93 ± 1.43^ab^	0.046
	30–60	36.91 ± 1.60^b^	37.88 ± 1.28^a^	37.60 ± 0.83^a^	<0.001
	0–60	36.68 ± 1.61^b^	37.51 ± 2.11^a^	37.26 ± 1.21^a^	<0.001
Feed intake	0–30	24.82 ± 2.46	24.17 ± 2.60	24.51 ± 2.50	0.072
	30–60	24.88 ± 2.48	24.29 ± 2.09	24.67 ± 2.87	0.069
	0–60	24.80 ± 2.96	24.23 ± 2.86	24.59 ± 2.70	0.063
Feed to milk ratio	0–30	0.67 ± 0.05^a^	0.65 ± 0.06^b^	0.66 ± 0.04^ab^	0.036
	30–60	0.67 ± 0.05^a^	0.64 ± 0.06^b^	0.65 ± 0.05^b^	0.042
	0–60	0.67 ± 0.05^a^	0.64 ± 0.06^b^	0.65 ± 0.05^b^	0.035

^a,b,c^Means within a row-column with no common superscripts differ significantly (*p* < 0.05).

### 3.2 Milk quality

Milk composition parameters (fat, protein, lactose, and solids-not-fat) were not statistically significant by musical intervention (Table 2).

**Table 2:** Effect of Raga and Chinese five-element music on milk quality.

Items	Time/d	Control	Raga	Chinese five-element	*P*
Milk fat percentage (g/100 g)	0	3.56 ± 0.31	3.70 ± 0.49	3.58 ± 0.39	0.476
	30	3.82 ± 0.43	3.89 ± 0.65	3.84 ± 0.34	0.896
	60	4.00 ± 0.56	4.16 ± 0.73	4.10 ± 0.53	0.701
Milk protein (g/100 g)	0	3.44 ± 0.25	3.48 ± 0.20	3.46 ± 0.25	0.965
	30	3.56 ± 0.22	3.60 ± 0.27	3.58 ± 0.26	0.872
	60	3.55 ± 0.22	3.56 ± 0.29	3.56 ± 0.33	0.988
Milk lactose (g/100 g)	0	5.29 ± 0.16	5.32 ± 0.18	5.29 ± 0.15	0.679
	30	5.38 ± 0.23	5.41 ± 0.17	5.39 ± 0.19	0.925
	60	5.35 ± 0.17	5.89 ± 0.48	5.38 ± 0.22	0.937
Total solids (g/100 g)	0	13.30 ± 1.02	13.29 ± 0.95	13.38 ± 0.91	0.951
	30	13.24 ± 0.93	13.30 ± 0.99	13.26 ± 0.51	0.974
	60	13.84 ± 1.05	14.10 ± 0.56	13.97 ± 0.82	0.624
SCC (10,000/mL)	0	41.65 ± 5.07	41.23 ± 6.44	41.21 ± 5.93	0.965
	30	46.25 ± 5.25	44.47 ± 5.39	44.76 ± 6.54	0.577
	60	43.09 ± 3.98	42.90 ± 5.55	43.25 ± 6.60	0.980
Non-fat solids (g/100 g)	0	9.30 ± 0.31	9.28 ± 0.47	9.31 ± 0.30	0.961
	30	9.45 ± 0.27	9.48 ± 0.32	9.47 ± 0.42	0.970
	60	9.43 ± 0.27	9.51 ± 0.25	9.46 ± 0.32	0.739

### 3.3 Serum biochemical indices

At d 30, GLU concentrations were significantly higher (*p* < 0.05) in the Raga treatment than in the control and Chinese five-element treatment (Table 3). By d 60, the Raga treatment maintained elevated GLU (*p* < 0.05) while exhibiting reduced alanine aminotransferase (ALT; *p* < 0.05) and increased albumin (ALB; *p* < 0.05) compared with controls. Additionally, triglyceride (TG) concentrations were markedly lower (*p* < 0.001) in the Raga treatment than in both other treatment at the end of the trial.

**Table 3:** Effect of Raga and Chinese five-element music on serum biochemical indices of dairy cows.

Project	Time/d	Control	Raga	Chinese five-element	*P*
ALT (U/L)	30	34.84 ± 6.17	36.88 ± 4.47	35.31 ± 6.32	0.549
	60	35.61 ± 6.11^a^	32.38 ± 3.77^b^	35.50 ± 4.21^a^	0.034
ALB (g/L)	30	35.10 ± 1.22	35.19 ± 1.07	35.40 ± 0.32	0.761
	60	34.67 ± 1.15^b^	35.58 ± 1.24^a^	34.75 ± 1.04^b^	0.040
TG (mmol/L)	30	0.57 ± 0.18	0.50 ± 0.09	0.52 ± 0.13	0.263
	60	0.59 ± 0.09^a^	0.46 ± 0.03^c^	0.52 ± 0.07^b^	<0.001
GLU (mmol/L)	30	1.51 ± 0.21^b^	1.75 ± 0.25^a^	1.52 ± 0.31^b^	0.015
	60	1.42 ± 0.25^b^	1.81 ± 0.30^a^	1.72 ± 0.27^a^	0.001
NEFA (mmol/L)	30	0.19 ± 0.05	0.17 ± 0.04	0.17 ± 0.03	0.481
	60	0.18 ± 0.04	0.16 ± 0.01	0.17 ± 0.03	0.153

ALT, alanine aminotransferase; ALB, serum albumin; TG, triglycerides; GLU, glucose; NEFA, free fatty acids.

^a,b^Means within a row-column with no common superscripts differ significantly (*P* < 0.05).

### 3.4 Serum antioxidant capacity

Cows exposed to music had greater GSH-PX activity (*p* < 0.001) and lower MDA concentrations (*p* < 0.001) than controls at d 30 (Table 4). These improvements in antioxidant status persisted through d 60, with both music treatment maintaining higher GSH-PX activity (*p* < 0.001) and reduced MDA levels (*p* < 0.001).

**Table 4:** Effect of Raga and Chinese five-element music on serum antioxidant indices of dairy cows.

Project	Time/d	Control	Raga	Chinese five-element	*P*
GSH-PX (U/mL)	30	9.12 ± 1.05^b^	11.61 ± 1.83^a^	9.56 ± 1.35^b^	<0.001
	60	9.02 ± 0.68^c^	10.78 ± 0.59^a^	10.14 ± 1.05^b^	<0.001
SOD (U/mL)	30	104.59 ± 19.29	116.04 ± 27.73	110.13 ± 23.12	0.361
	60	104.52 ± 19.92	117.61 ± 29.27	110.12 ± 21.91	0.315
T-AOC (μmol/mL)	30	2.46 ± 0.32	2.68 ± 0.09	2.60 ± 0.24	0.063
	60	2.37 ± 0.32	2.44 ± 0.30	2.27 ± 0.30	0.272
MDA (nmol/mL)	30	23.76 ± 2.78^a^	19.51 ± 2.81^b^	19.59 ± 3.98^b^	<0.001
	60	23.04 ± 3.79^a^	18.25 ± 1.73^c^	20.90 ± 2.00^b^	<0.001

GSH-Px, glutathione peroxidase; SOD, superoxide dismutase; MDA, malondialdehyde; MDA, malondialdehyde.

^a,b,c^Means within a row-column with no common superscripts differ significantly (*P* < 0.05).

### 3.5 Immune function

By d 30, Raga-exposed cows had higher serum IgA than both other treatment (*p* < 0.05) and greater IgM than controls (*p* < 0.05; Table 5). These immunomodulatory effects continued at d 60, with both music treatment showing elevated IgG and IgM (*p* < 0.05) compared with controls.

**Table 5:** Effect of Raga and Chinese five-element music on serum immunity indexes of dairy cows.

Project	Time/d	Control	Raga	Chinese five-element	*P*
IgG (μg/mL)	30	155.83 ± 20.08	170.45 ± 24.14	163.9 ± 34.14	0.283
	60	155.89 ± 23.58^b^	187.50 ± 23.57^a^	185.83 ± 35.66^a^	0.002
IgA (μg/mL)	30	15.66 ± 3.51^b^	19.88 ± 4.64^a^	18.68 ± 3.09^a^	0.007
	60	12.90 ± 3.91	15.02 ± 4.25	14.38 ± 4.79	0.355
IgM (μg/mL)	30	10.21 ± 1.14^b^	11.37 ± 1.61^a^	10.96 ± 1.18^ab^	0.034
	60	9.74 ± 1.47^b^	11.71 ± 1.47^a^	11.61 ± 2.22^a^	0.002

IgA, immunoglobulin A; IgM, immunoglobulin M; IgG, immunoglobulin G.

^a,b^Means within a row-column with no common superscripts differ significantly (*P* < 0.05).

### 3.6 Neuroendocrine index

At d 30, the Raga treatment significantly modulated neuroendocrine and stress-related markers (Table 6): Glu and COR were decreased (*p* < 0.05 and *p* < 0.001, respectively); GH, 5-HT, β-EP, SP, and GABA were increased (*p* < 0.05 to *p* < 0.001).

**Table 6:** Effect of Raga and Chinese five-element music on serum neuroendocrine index in dairy cows.

Project	Time/d	Control	Raga	Chinese five-element	*P*
Glu (μmol/L)	30	9.66 ± 1.07^a^	8.40 ± 1.23^b^	9.13 ± 0.99^ab^	0.005
	60	8.98 ± 0.78^a^	8.13 ± 0.58^b^	8.40 ± 0.64^b^	0.002
GH (μg/L)	30	7.26 ± 1.76^b^	9.24 ± 1.30^a^	8.45 ± 1.09^a^	0.001
	60	5.61 ± 0.56^b^	6.38 ± 1.00^a^	5.96 ± 0.97^ab^	0.044
COR (μg/L)	30	44.13 ± 9.55^a^	33.21 ± 1.91^b^	34.01 ± 9.55^b^	<0.001
	60	32.41 ± 7.07^a^	22.37 ± 1.91^b^	24.24 ± 3.11^b^	<0.001
5-HT (ng/L)	30	170.52 ± 11.36^b^	183.09 ± 12.59^a^	180.00 ± 13.51^a^	0.014
	60	174.13 ± 15.77^b^	189.60 ± 17.52^a^	188.40 ± 16.20^a^	0.019
β-EP (ng/L)	30	14.42 ± 1.34^b^	16.30 ± 1.43^a^	15.30 ± 1.15^b^	<0.001
	60	14.17 ± 1.02^b^	15.55 ± 1.36^a^	14.67 ± 1.03^b^	0.004
SP (ng/L)	30	35.48 ± 3.21^b^	39.14 ± 2.93^a^	36.27 ± 2.37^b^	0.002
	60	32.59 ± 2.49^b^	35.96 ± 2.48^a^	34.18 ± 2.79^ab^	0.002
GABA (μmol/L)	30	6.20 ± 0.49^b^	6.77 ± 0.46^a^	6.40 ± 0.52^b^	0.010
	60	5.95 ± 0.54^b^	6.48 ± 0.60^a^	6.28 ± 0.63^a^	0.029

Glu, glutamate; GH, growth hormone; COR, cortisol; 5-HT, 5-hydroxytryptamine; β-EP, β-endorphin; SP, substance P; GABA, gamma-aminobutyric acid.

^a,b^Means within a row-column with no common superscripts differ significantly (*P* < 0.05).

### 3.7 Welfare indicators

Cows in the Raga treatment displayed reduced activity levels (*p* < 0.05) compared with both other treatment during d 30 to 60 (Table 7). Over the entire experimental period, activity was lower (*p* < 0.05) in the Raga treatment than in controls.

**Table 7:** Effect of Raga and Chinese five-element on welfare indicators of dairy cows (min).

Project	Time/d	Control	Raga	Chinese five-element	*P*
Activity level	0–30	504.73 ± 43.97	485.23 ± 46.94	489.95 ± 25.89	0.190
	30–60	492.55 ± 40.88^a^	453.33 ± 33.17^b^	471.13 ± 29.86^a^	0.014
	0–60	498.64 ± 42.26^a^	469 ± 43.29^b^	480.54 ± 29.15^ab^	0.014
Rumination time	0–30	508.05 ± 30.35	510.04 ± 57.12	503.11 ± 39.60	0.905
	30–60	489.72 ± 30.84	505.66 ± 43.18	508.58 ± 46.87	0.363
	0–60	498.61 ± 31.53	507.85 ± 49.73	506.01 ± 43.01	0.649
Lying time	0–30	597.71 ± 35.70	600.05 ± 67.20	591.89 ± 46.59	0.905
	30–60	576.15 ± 36.29	594.90 ± 50.80	598.33 ± 55.14	0.363
	0–60	586.60 ± 37.09	597. 47 ± 58.51	595.31 ± 50.60	0.649

^a,b^Means within a row-column with no common superscripts differ significantly (*P* < 0.05).

## 4 Discussion

Numerous studies have demonstrated that music therapy can enhance animal production performance. Cows exhibit distinct physiological responses to different types of music, with some music positively influencing milk production while others have the opposite effect. For instance, a study has shown that playing classical music can increase milk production in cows by 5–15% and improve feed efficiency, as evidenced by an 8–12% reduction in the feed conversion ratio (11). Carnatic music can effectively enhance milk production in dairy cows, particularly increasing it by 12–18% during the winter season (20). Exposure to musical stimuli increased milk production in dairy cows by 13.2% and reduced residual milk yield (21). Soft music also improved milk production, with the classical music group showing the highest milk yield, an increase of 7–10% compared to the control group, followed by pop music (an increase of 4–6%) (15), but rock music reduces milk production (14). It has also been found that milk production in the treatment without music was slightly higher than in the treatment with music 6–12% (22). In this experiment, statistical analysis revealed that the milk production of cows exposed to Raga music and Chinese five-element music was higher than that of the control group. This demonstrates that Raga music and Chinese five-element music positively correlate with milk yield in cows and can help increase milk production. From the feed-to-milk ratio perspective, Rage music group dairy cows had a lower feed conversion rate than Chinese five-element music group ones, crucial for efficiency-focused milk factories. Raga music is characterized by a gentle rhythm, consisting only of tones with no lyrics. The tone of the music is gentle and calm, which can significantly soothe emotions (17). The characteristics of Chinese five-element music are similar to Raga music, which has a slow tempo and frequency and improves cows’ milk production. By playing these two types of music, noise pollution caused by intensive production may have been reduced, creating a relaxing and comfortable environment for the cows, thus increasing their milk production. Milk quality is associated with mastitis in dairy cows (23). However, we found no effect of music on milk quality. We speculate that changes in milk quality require adequate supply of specific nutrients. However, feed intake and formulation were consistent across the three groups of cows in this trial, so no changes in milk components occurred. Additionally, some researchers have found that music does not affect milk fat and protein percentages (21). The study revealed that the feed-to-milk ratio was reduced in cows exposed to music. It was hypothesized that the selected music can enhance digestion and nutrient absorption, thereby increasing milk yield. This effect may be attributed to music-induced comfort, which reduced physical activity, prolonged rumination time, and improved digestive efficiency. These findings align with subsequently measured welfare indicators.

Serum biochemical indicators reflect an organism’s nutritional metabolism, stress, and overall health status. GLU serves as the primary energy source for vital physiological activities, and fluctuations in GLU levels can provide insight into an animal’s sugar metabolism. Additionally, lipid metabolism efficiency can be evaluated based on the levels of TG and NEFA; lower TG and NEFA levels generally indicate improved lipid utilization (24–27). ALB is predominantly synthesized in the liver, and its serum concentration is an essential marker of liver function. A significant decrease in ALB levels is commonly associated with substantial liver damage (28). Similarly, alanine ALT is a sensitive indicator of liver cell membrane integrity, with elevated ALT levels signaling more significant hepatocellular damage (29). Latin music increases the total protein content in serum by 5%, while African percussion music reduces triglycerides by 8% (30). In this study, cows exposed to Raga music exhibited lower ALT levels, GLU and ALB concentrations, and reduced TG levels. We hypothesized that Raga music has a stronger effect than Chinese five-element music, potentially due to its complex improvisational rhythmic structures and microtonal scales that dynamically interact with the auditory system, whereas Chinese five-element music typically relies on simple pentatonic melodies and fixed frequency ratios aligned with natural rhythms, leading to less pronounced effects.

For dairy cows, the stage of increasing milk production is particularly critical, but it may trigger a series of health issues, such as mastitis, reduced reproductive performance, heat stress syndrome, and metabolic diseases. Meanwhile, it significantly decreases feed conversion efficiency and milk yield. Therefore, alleviating oxidative stress is a key link to maintain the health of dairy cows, optimize production performance, and enhance breeding economic benefits (31). GSH-Px and SOD are crucial roles in combating oxidative damage. MDA is a product of lipid peroxidation and serves as an indicator of the state of oxidative stress. The content of T-AOC reflects the metabolism of free radicals in the animal’s body (32). Previous studies have shown that playing classical music alleviates oxidative stress in broilers housed under high-density conditions (13). Turkish classical music also benefits patients’ pain and oxidative stress (33). These findings are consistent with our results. The serum levels of GSH-PX, SOD, and T-AOC were higher, and the levels of MDA were lower in cows in the music treatment compared to the control group. Previous research has also indicated that playing music reduces stressful behaviors and alleviates animal stress and anxiety (34, 35).

In animals, IgA and IgG are core molecules of immune defense, our previous studies have shown that playing classical music for broilers increases serum IgA and IgG levels, improving their immune function (13). A separate study found that music enhanced rodents’ immune function and reduced the incidence of allergic reactions (8). IgA levels were found to increase after exposure to music, with only one study reporting a significant decrease in IgA levels (1). Music may indirectly regulate the immune system by influencing the neuroendocrine system, maintaining immune homeostasis through reducing stress hormone levels (36). Classical music reduces stress and enhances immune cell function in laying hens (37). In the present study, both Raga music and Chinese five-element music were found to increase IgM, IgG and IgA levels in cows. We hypothesize that music may enhance their immunity by modulating the nervous system and reducing stress levels.

Mindfulness training with musical accompaniment can effectively mitigate stress and reduce cortisol levels in students through a dual-pathway mechanism that regulates the hypothalamic–pituitary–adrenal axis (involving cortisol) and the autonomic nervous system (38). It can also decrease COR levels and increase growth hormone (GH) levels during surgery, as COR reflects the stress level and immune function of dairy cows, whereas GH is associated with lactation (39). Slow-paced, soft music, in particular, helps reduce arousal levels, promote relaxation, and alleviate anxiety (1, 36, 40). Additionally, music can mitigate fluctuations in blood pressure, heart rate, and both sympathetic and parasympathetic nervous activity, thereby lowering blood COR levels in rodents (8). Studies have found that dopamine levels in the nucleus accumbens and dorsal striatum of rats in the melodic music group significantly increased. Melodic music may activate dopamine in the reward pathway and 5-HT in the emotion regulation pathway, increasing the latter by 18%, thereby influencing motivation, motor control, and emotion-related behaviors (41). In our study, cows in the Raga music treatment exhibited significantly lower blood concentrations of Glu and COR compared to the control group while demonstrating elevated levels of GH, 5-HT, *β*-EP, and GABA. These findings indicate that dairy cows in intensive farming systems are more susceptible to physiological stress responses. Previous studies have demonstrated that music therapy may ameliorate stress by regulating amino acid neurotransmitters, reducing Glu secretion and increasing acid GABA levels (42). As 5-HT and GABA are used to evaluate neuroendocrine balance and monitor nervous system stability, playing soft music for cows has been found to raise blood GABA levels, promote GH secretion, and increase 5-HT content (15). The study also concluded that music increased the serotonin level in dairy cows’ blood (21). Therefore, Indian Raga and Chinese five-element music have been shown to elevate related hormones in cows, which aligns with previous studies and contributes to the gap in research on Chinese five-element music in dairy cows. While our current study has provided valuable insights, further research is needed to understand better the mechanisms through which music exerts its effects.

Improving animal welfare is crucial for ensuring both the physiological and psychological health of animals and, to some extent, can significantly enhance the productivity of farmed animals (43). A study found that cows were calmer when classical music was played, while they exhibited more agitation when reggae music was played (22). In dairy farming, playing soothing music (60–80 bpm) reduces restlessness during milking by 18% and prolongs lying time by 15% (44), this phenomenon is directly associated with increased rumination time after feeding—a key indicator positively correlated with lying duration and used to assess digestive health and psychological relaxation in cows (45). Notably, such behavioral improvements have a clear physiological basis: when milking facility noise (e.g., equipment vibration, mechanical sounds) elevates cortisol levels in cows, their standing restlessness increases by 25% (46). Music reverses this trend through sound pressure neutralization, reducing animal activity below baseline levels. (47). Reducing ecological stress can improve the feed efficiency of dairy cows (48). For example, playing music for piglets has been shown to reduce post-weaning aggression, increase resting time, and improve piglet weight after weaning (49). In other animal studies, piglets subjected to conditioned reflex training combining playtime-music showed a 28% reduction in the incidence of stereotypic behaviors and promoted sustained group lying (11). In this study, we observed that Raga music and Chinese five-element music reduced the activity levels of cows and increased their lying time. We hypothesize that these two types of music alleviate environmental stress in cows in intensive production systems by modulating the levels of stress hormones in their bloodstream, thereby enhancing their comfort and welfare. These findings are consistent with the results of previous studies. Overall, music therapy holds significant potential for improving animal welfare, alleviating stress, and enhancing production efficiency through the modulation of physiological rhythms and psychological balance (17).

Although we observed that dairy cows in the music intervention group showed increased milk production, enhanced antioxidant capacity, and alleviated stress and inflammatory responses, multiple factors need to be considered in practical production. For example, this experiment was conducted in spring (March to May) at an average temperature of 15°C, without multi-seasonal trials or long-term cycle tests. Additionally, the study overlooked the impacts of climatic factors such as air humidity and environmental noise interference, as well as whether dairy cows might develop adaptability to repeated musical stimulation.

## 5 Conclusion

Studies have indicated that exposure to music may improve milk production in dairy cows, enhance their antioxidant capacity, and mitigate stress and inflammatory responses. However, comprehensive indicators show that Raga music is more effective than Chinese five-element music in achieving these effects. These findings suggest that Raga music may alleviate oxidative stress induced by noise pollution in intensive farming systems. While these findings support the potential of music as a non-invasive welfare-enhancing tool, further long-term and large-scale studies are needed to confirm these effects and to understand the underlying mechanisms.

## Data Availability

The raw data supporting the conclusions of this article will be made available by the authors, without undue reservation.
