# Supplementary material for: Effects of Raga music and Chinese five-element on milk production, antioxidant, neuroendocrine, immune, and welfare indicators in dairy cows
Source: Front Vet Sci. 2025 Jun 30;12:1623026. doi: 10.3389/fvets.2025.1623026 (PMC12256249; doi:10.3389/fvets.2025.1623026)
Supplement: Supplementary file 1 [file Table_1.DOCX]

**Table 1.** Experiment music list

| Raga Music | TCM five elements music |
| --- | --- |
| Ba hudari | Bloom-Heart Wellness Fun |
| Brova Barama (Kriti); Ragam | Breaking the Cocoon - Liver Wellness Fun |
| Kanada | Harmony - Spleen Wellness Fun |
| Mamava Sada (Kriti); Ragam | Yun Ning - Lung Nutritional Joy |
| Raag Yaman | Heavenly Stream - Kidney Health Fun |
| Indian Raga Shyam Kalyan | Yang Rhyme-Xuan Tian Warm Wind |
| Shehnai1 | Yin Rhyme-Blue Leaves and Smokey Clouds |
| Shehnai2 | Yin Rhyme - Rainbow After Rain |
| Veena Concert | Yang Yun-Lotus Reflecting the Sun |
| Sitar | Yang Yun-Lotus Reflecting the Sun |

**Table 2.** Diet composition and nutrient content (% dry basis)

| Dietary material composition | Form | Nutrition level | Content |
| --- | --- | --- | --- |
| Corn Silage | 58.4 | Crude fat | 6.35 |
| Corn flour | 11.5 | Crude protein | 16.14 |
| Steam-flaked corn | 3.5 | Neutral detergent fiber | 37.24 |
| double-low rapeseed meal | 7.4 | Acid detergent fiber | 21.05 |
| Premix | 6.6 | Calcium | 0.94 |
| Cottonseed fluff | 5.2 | Phosphorus | 0.48 |
| Corn Silage | 4.2 |  |  |
| Soybean meal | 2.1 |  |  |
| Extruded soybean | 1.1 |  |  |
| Total | 100 |  |  |
